# Advancement in nanomaterials for environmental pollutants remediation: a systematic review on bibliometrics analysis, material types, synthesis pathways, and related mechanisms

**DOI:** 10.1186/s12951-023-02151-3

**Published:** 2024-01-10

**Authors:** Nosheen Asghar, Alamdar Hussain, Duc Anh Nguyen, Salar Ali, Ishtiaque Hussain, Aurangzeb Junejo, Attarad Ali

**Affiliations:** 1 Department of Global Smart City, Sungkyunkwan University (SKKU), 2066 Seobu-ro, Jangan-gu, Suwon, Gyeonggi-do 16419 Republic of Korea; 2 Department of Botany, University of Baltistan, Skardu 16400, Gilgit-Baltistan, Pakistan; 3 Department of Environmental Science, University of Baltistan, Skardu 16400, Gilgit-Baltistan, Pakistan; 4 Department of Environmental Science, Quaid-i-Azam University of Islamabad, Islamabad, 15320 Pakistan; 5 Directorate of Quality Enhancement Cell, University of Baltistan, Skardu 16400, Gilgit-Baltistan, Pakistan

**Keywords:** Environmental contaminations, Wastewater treatment, Nanocomposites, Adsorption

## Abstract

Environmental pollution is a major issue that requires effective solutions. Nanomaterials (NMs) have emerged as promising candidates for pollution remediation due to their unique properties. This review paper provides a systematic analysis of the potential of NMs for environmental pollution remediation compared to conventional techniques. It elaborates on several aspects, including conventional and advanced techniques for removing pollutants, classification of NMs (organic, inorganic, and composite base). The efficiency of NMs in remediation of pollutants depends on their dispersion and retention, with each type of NM having different advantages and disadvantages. Various synthesis pathways for NMs, including traditional synthesis (chemical and physical) and biological synthesis pathways, mechanisms of reaction for pollutants removal using NMs, such as adsorption, filtration, disinfection, photocatalysis, and oxidation, also are evaluated. Additionally, this review presents suggestions for future investigation strategies to improve the efficacy of NMs in environmental remediation. The research so far provides strong evidence that NMs could effectively remove contaminants and may be valuable assets for various industrial purposes. However, further research and development are necessary to fully realize this potential, such as exploring new synthesis pathways and improving the dispersion and retention of NMs in the environment. Furthermore, there is a need to compare the efficacy of different types of NMs for remediating specific pollutants. Overall, this review highlights the immense potential of NMs for mitigating environmental pollutants and calls for more research in this direction.

**Graphical Abstract:**

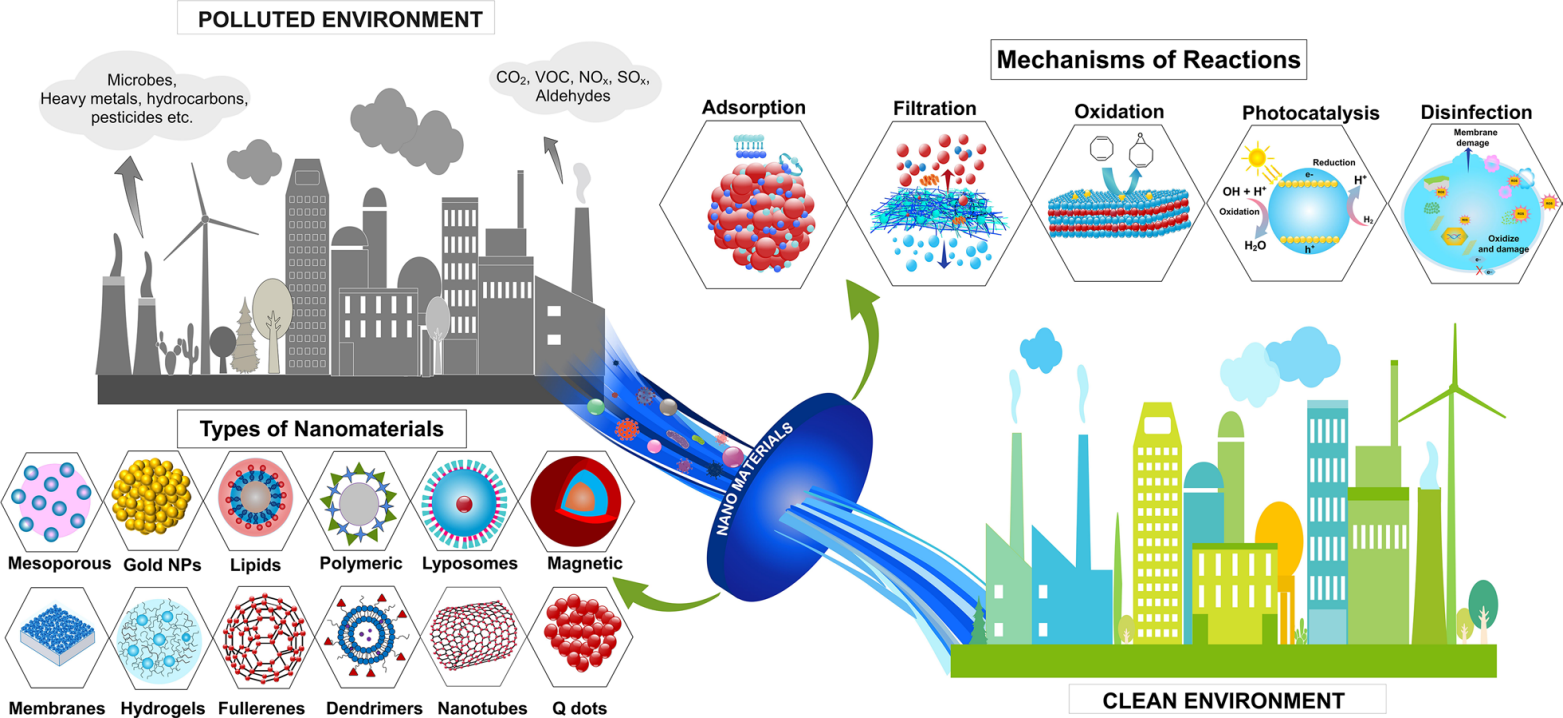

## Introduction

Exponential population growth and rapid global industrialization result in a significant discharge of pollutants into the environment. Environmental contamination has become a major issue worldwide because even a small concentration of toxic pollutants can lead to serious health issues for human and animals [1, 2]. Undesirable gases, bioaerosols, oxides, microbes, sooth, heavy metals, and other toxic materials in outdoor and indoor air cause severe effects on human health and environment [3]. According to the World Health Organization (WHO), approximately 91% of the world's population resides in places where pollutant levels exceed thresholds of WHO air quality guidelines. Ambient air pollution is estimated to cause 4.2 million premature deaths globally [4]. Additionally, millions of harmful effluents in water from industrialization and urbanization effect the water quality [1, 5] Those pollutants mainly include pharmaceuticals, metals, dyes, pesticides, fertilizers, microorganisms (MOs), personal care products and radionuclides, etc., are significant threats towards global water security [6–8]. Those pollutants enter the food chain and cause detrimental effects on human health and negatively affect socio-economic development [9]. Thus, it is crucial to determine and eliminate primary sources and concentrations of all contaminants using cutting-edge technologies that could deliver reliability and high quality cost-effectively and comply with environmental standards and regulations [8, 10]. Numerous remediation techniques have been developed during the last few decades to address air and water contaminants, including physical, chemical, and biological methods; however, most of those treatment techniques have significant limitations, such as high costs, the complexity of the operation, and secondary contamination [11].

**Fig. 1:**
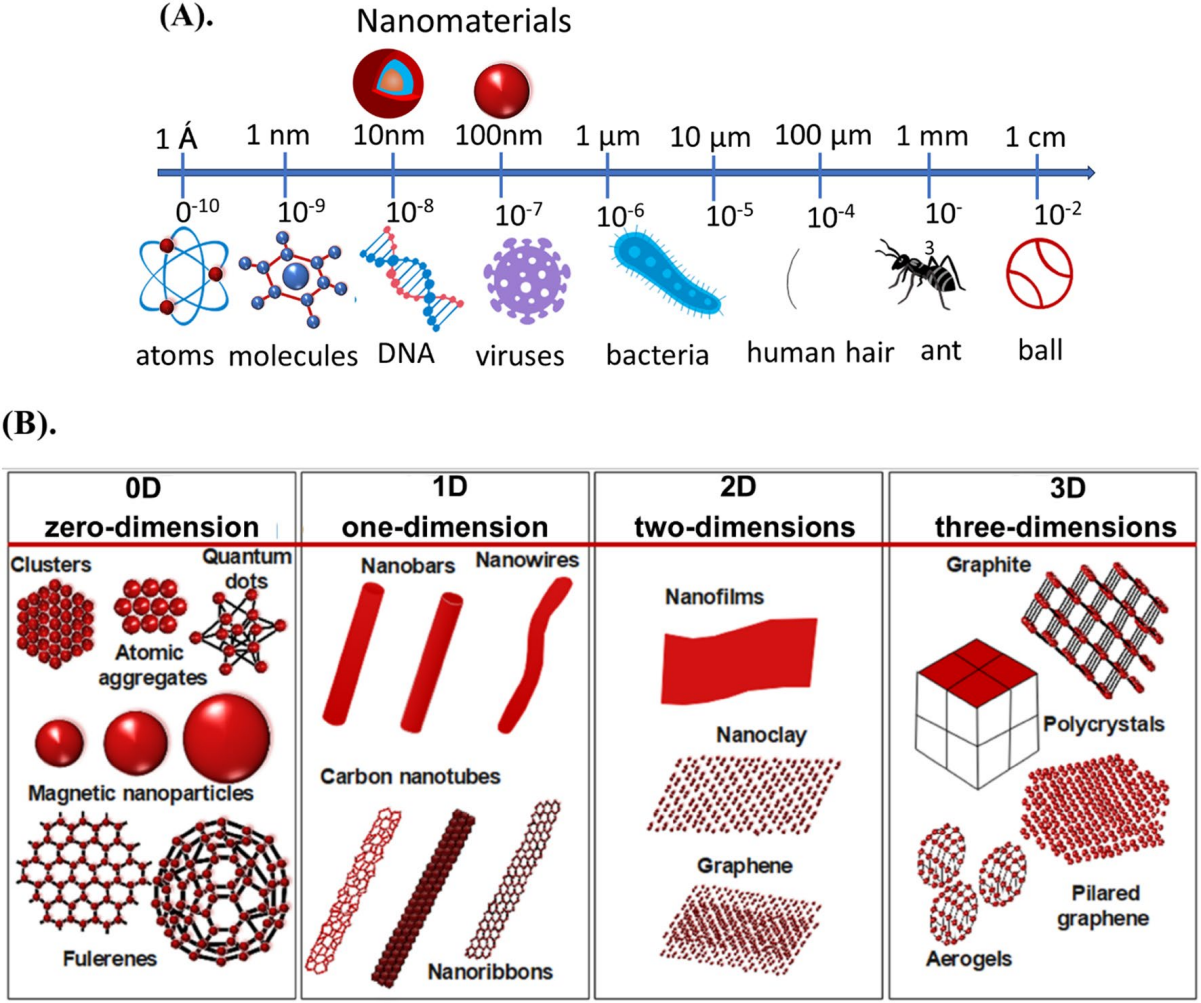
**A** Comparison of size of nanomaterial with common materials **B** schematic illustration of low-dimensional nanostructures: zero-dimension (0D), one-dimension (1D), two-dimensions (2D), and three-dimensions (3D). Information was adapted and modify from **A** [22] **B** [23]

Nanomaterials (NMs) are ultrafine particles ranging from 1 to 100 nm though size does not give a satisfactory definition of NMs classification as shown in Fig. 1A. They can be natural, manmade, or incidental materials [12]. NMs exhibit diverse structural dimensions, including zero-dimensional (0D), one-dimensional (1D), two-dimensional (2D), and three-dimensional (3D) arrangements. In the 0D configuration, nanometer-scale dimensions are observed along all three axes (x, y, z). In the case of 1D materials, nanometer-scale features are limited to two dimensions. Similarly, 2D structures possess nanometer-scale attributes in just one direction. Notably, the classification extends to encompass three-dimensional (3D) nanostructures, despite their dimensions exceeding 100 nm as shown in Fig. 1B. NMs can be synthesized through chemical, biological, and green-based routes [3]. During the past few years NMs are gaining much interest in environmental applications as promising adsorbents and catalysts for the application of environmental remediation [13]. Through their unique redox properties and significant features such as size, dissolution/solubility, surface area, surface charge, and surface chemical composition beneficial for the removal of redox-sensitive pollutants via degradation [14, 15]. Many types of NMs have been developed to treat water contaminations and air cleaning as a cost-effective and reliable technique because they provide high adsorption capacities and increased surface area compared to micro and macrostructures [16, 17]. Additionally, careful tunning and surface modification of NMs provides additional characteristics and substantial benefits for tackling environmental contamination. Some NMs eliminate, while others sequester pollutants [18]. However, NMs have some drawbacks, such as high costs, potential toxicity, challenges with recycling, and interactions with other media. Moreover, exposure and unintentional release of NMs pose substantial risks and health concerns [19]. Several dependable, low-cost, and environmentally friendly NMs with various functions have been described for detoxifying pollutants from air and water [5, 20, 21]. However, it should be safe and demonstrate strong sorption capacity and selectivity, particularly because pollutants found in low concentrations should easily be removed and recycled. Much research in recent years has proven that NMs can meet most of those requirements.

Bibliometrics is a helpful tool that offers direction for ongoing research and future studies worldwide. It uses quantitative and statistical analysis to explain how research papers are distributed within a particular topic, field, institution, and nation. They can offer a more thorough analysis to show research fields, development patterns or direction. Even though there has been significant growth in NMs research on remediation of air and water pollution, however, a comprehensive and systematic review on bibliometrics, NMs synthesis pathways, and reaction mechanism is still absent. Therefore, in this review paper, we investigated and analyzed literature in the past 10 years to better understand NMs application and research progress in air and water pollution treatment. Furthermore, we systematically discussed conventional and advanced technologies for the remediation of contaminants from air and water. Additionally, a brief overview of the importance of NMs, types, synthesis pathways, pros and cons, and mechanisms of reactions were discussed thoroughly. Finally, some shortcomings and recommendations of NMs for pollutant remediation were mentioned for future research directions.

## Bibliometric analysis

**Fig. 2:**
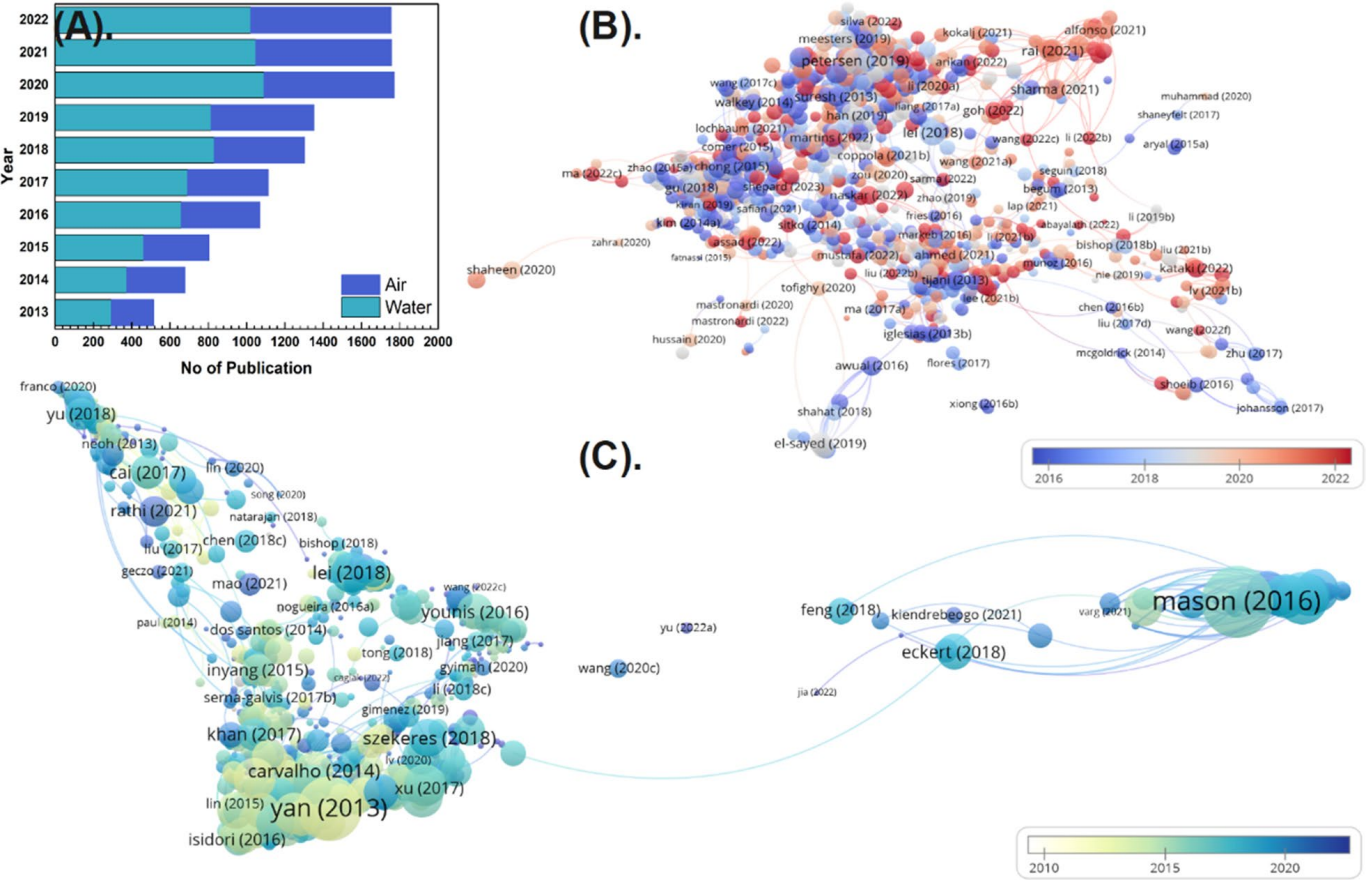
**A** Increase of studies on remediation of air and water pollution using nanomaterials from 2013 to 2022 **B** Distribution of studies using nanomaterials for air pollution remediation, and **C** Distribution of studies using nanomaterials for water pollution remediation (Data taken from Web of Science)

The basic bibliometrics of research papers on air and water pollution remediation using NMs during 2013–2022 are shown in Fig. 2A. The results show that the number of related research papers on air pollution treatment using NMs has rapidly increased yearly from 2013 (224) to 2022 (732). A similar trend was observed for wastewater treatment using NMs in the last 10 years, with the total number of publications in 2013 (292) increasing to (1022) in 2022. It slightly decreases from 2020 to 2021 due to the corona pandemic. In this review, we follow a similar methodology to other bibliometric studies. Data were obtained from Thomson Reuters online science citation index (SCI) expanded databases of the web of science on 17th May 2022. Bibliometric analysis was done by searching in "Web of Science "for the words "Nanomaterial" (topic) and "air pollution treatment" (topic), and "wastewater treatment" (topic). Ten high-impact factors well famous international journals were included in our bibliometric analysis. Only journal research articles from top relevant journals related to NMs usage for the treatment of air and water pollution have been compiled for the bibliography. All the details of the articles, including title, year of publication, keywords, abstract, funding agencies, web of science categories of the article, and names of journals, were transferred into a spread excel sheet. VOS viewer software for creating and displaying bibliometric networks was used [24]. The affiliation of at least one author to the articles served as a proxy for the contributions of various institutions and nations, and the phrase "single country article" was applied when the researchers' addresses were from the same nation. Cite space 5 was applied in the co-citation analysis. The most co-cited article was thought to be the most populous work in this field and thought to be a pioneer or hot issue. Co-citation and author keyword analyses were conducted to identify the current hot topics and significant research trends. Co-citation occurs when two works are both cited in the same work. According to co-citation theory, the strength of co-citation between cited articles reflects their inherent association. Figure 2B and C presents the co-citation cluster's outcome of air and water pollution remediation. This analysis gives us a historical perspective on the development of intensive scientific research on a specific topic of sciences has over 100 branches in different cities, and articles divided into branches and would result in a different ranking.

## Remediation strategies

### Conventional strategies

A wide range of treatment technologies has been developed to minimize the concentration of pollutants in the environment. These technologies are crucial in reducing the negative impact of pollution on the environment and human health. However, despite various treatment methods, their effectiveness varies, and they often have limitations such as low stability and high complexity [25]. In air treatment, several strategies, such as activated carbon adsorption and selective catalytic reduction, wet flue gas desulfurization (WFGD), and activated carbon injection (ACI), have been utilized to reduce the concentration of pollutants [26, 27]. Likewise, water treatment techniques involve chemical, physical, thermal, mechanical, and biological methods [5, 28, 29]. Further techniques include wet oxidation, electrocoagulation, ion exchange, ozonolysis, Fenton, adsorption, extraction, flocculation/coagulation, evaporation, steam stripping, distillation, filtration, floatation, screening, sedimentation, reverse osmosis, forward osmosis, phytoremediation, bioaccumulation, biotransformation, and biomineralization [5, 30–32]. However, they often involve high capital and operational costs, and their efficiency is limited by membrane fouling, low selectivity, and the disposal of residual sludge [8, 30, 33]. To address the limitations of conventional treatment methods, researchers worldwide have focused on developing NMs as an alternative approach with strong oxidation power that can oxidize and mineralize various organic and inorganic contaminants. NMs have shown promising results in pollutant removal and have the potential to provide a low-cost and environmentally acceptable solution. Therefore, developing and implementing NMs in treatment processes can potentially revolutionize pollution control and management [26, 27].

### Nanomaterial-based strategies

#### Inorganic nanomaterials

##### Metal and metal oxide-based nanomaterials

Metal and metal oxide based NMs are some of the most widely used inorganic NMs for removing various hazardous pollutants from the environment. All three variants of iron oxide, namely magnetite (Fe_3_O_4_), maghemite (γ-Fe_2_O_3_), and hematite (α-Fe_2_O_3_), have been thoroughly examined for their suitability in pollution treatment applications. However, among them the Fe_3_O_4_, and γ-Fe_2_O_3_ are metallic in nature [34]. The commonly used metal oxide NM is nano zerovalent iron (NZVI), which contains a shell of Fe(II), Fe(III), and zerovalent iron and an outer mixed iron oxides shell layer [35]. Nanocomposites (NCs) of metals and magnetic iron oxides such as Fe_3_O_4_, zinc iron oxide (ZnFe_2_O_4_), unidentified iron oxide (uFe_2_O_4_), manganese iron oxide (MnFe_2_O_4_), and cobalt iron oxide (CoFe_2_O_4_) have also been extensively studied for the removal of pollutants especially heavy metals from the environment (refer to Tables 1 and 2) due to their magnetic properties, excellent separation, recyclability, high specific surface areas, high adsorption, and high binding energies [36]. The magnetic iron oxide based NMs can efficiently disperse in water and can be quickly recovered by an external magnetic field. The ability to recycle and reuse are the most important parameters. However, their real applications are limited by aggregation and oxidation, and their magnetic phase can still leak in acidic environments [26]. Aggregation of magnetic iron oxide base NMs alters the magnetic properties and reduces surface energy [34]. Thus, the functionalization of iron oxide NMs with inorganic and organic material having adsorption properties are recommended in the literature because they prevent iron oxide from oxidation [17, 37]. Surface functionalization with functional groups like carboxylic group (–COOH) and (–NH_2_) groups has been observed to confer notable enhancements in terms of stability, adsorption efficiency, and surface area especially for chelating metal ions [34]. Furthermore, magnetic iron oxide NMs exhibit the distinctive advantage of being recoverable, regenerable, and reusable subsequent to their application in diverse contexts. The recovery of these NMs through the implementation of magnetic field is extensively reported in relevant literature [38]. Additionally, separation and regeneration techniques for NMs include thermal separation and pH adjustment depending on type of coating material or functional groups [39].

**Table 1:** Summary of mechanisms and removal efficiency of different organic and inorganic nanomaterials for air pollutants remediation

Nanomaterials	Air pollutants	Mechanism	Removal efficiency (%)	References
CuO–MnO_2_–Fe_2_O_3_/ γ-Al_2_O_3_	Mercury (Hg°)	Thermal desorption	–	[109]
Cerium oxide	CO	Catalytic oxidation	100	[110]
Fe/Co co-doped/Mn-Ce/TiO_2_	NO and Hg°	Reduction and oxidation	55–92	[111]
Ti-doped Fe_3_O_4_ (1 1 1)	NOx	Catalytic oxidation	80	[112]
Silver, zinc, and iron	E. coli	Disinfection	97 ~ 99	[113]
Pt-TiO_2_	NOx	Catalysis	96.7	[114]
Graphene oxide	PM 2.5	Filtration	99	[58]
CoFe_2_O_4_-peroxymonosulfate	Hg°	Catalysis	85	[115]
Fe_3_O_4_@EDTA@Fe (II)	NOx	Adsorption	90	[116]
Iron-loaded ZSM-5 zeolite	SO_2_, NO, Hg°	Catalysis	100, 72.6, 93.4	[117]
Silver/polyacrylonitrile	Bacteria (E. coli)	Filtration	104 CFU/mL	[118]
ZnO	H_2_S	Adsorption	29.50 mg/g	[119]
bismuth oxide with graphene	Xylene	Photocatalysis	38.8–98.7	[120]
MnOx- MIL-100(Fe)	Hg°	Adsorption and oxidation	77.4	[121]
Silica(HS-UVM7-NH_2_-UVM7)	Lead (Pb)	Adsorption	95	[122]
MOF-801 and Cu_2_O	PM2.5 and PM10	Filtration, adsorption	64–85	[123]
Nd (neodymium) -TiO_2_	VOCs	Photocatalysis	60–80	[124]
V_2_O_5_-WO_3_/TiO_2_	NOx and Hg°	Catalytic reduction	93 ~ 99	[26, 125]
Thiol modified silica	Vanadium (V)	Adsorption	95	[126]
Polyacrylonitrile-boehmite	PM 2.5	Filtration	99.97	[127]
Hypochlorite (ClO^−^)	Sulfur gas	Adsorption	–	[26]
Nd (neodymium) -TiO_2_	NOx, VOCs, bioaerosols	Photocatalysis	60 ~ 80	[124]
Ca-doped ZnO	Tetracycline	Mineralization	99	[128]
Li_2_MnO_3_	CO, CO_2_	Chemisorption, Catalysis	–	[129]
Polysaccharides/MnO_2_-polymer fiber	Formaldehyde	Oxidation, catalysis	95.5	[130]

**Table 2:** Summary of experimental conditions, adsorption kinetic, removal efficiencies, and mechanisms of different organic and inorganic NMs for environmental pollutants remediation

Nanomaterials	Pollutants	Experimental conditions					q_max_ (mg/g) Ads kinetics	Removal efficiency(L/F^a^)	R^2^	Mechanism^b^	References
		Sorbent mg/L _initial_	Ads(g/L)	Temp (C)	pH _optimal_	Time (h)					
Fly ash	Methyl blue	16	0.01 mol/L	20	4.8–5.3	1	12.78 2nd	L	0.99	Co	[6]
OMWCCNTs	Pefloxacin, Cu (II)	50	1/0.2	25	3.5	4	204.4, 186.5 2nd	28.72 L	0.94	E, π–π, C	[131]
CS/MWCNT	Phenol	30	50/0.3	25, 40, 54	3–5	1	86.96 g/g 2nd	L	0.99	E, π–π	[60]
Co_2_Fe_2_O_4_@SiO2@EDTA	Hg	20	0.01	25	7	6	103.13 2^nd^	L	0.99	C	[132]
Fe_3_O_4_-C-MnO_2_	Uranium (U) (VI), europium (III)	100/0.05	0.3	25	5		77.71–51.01, 2nd	97.6	–	S.C	[133]
γ-Fe_2_O_3_	Vanadium	25,470	60	25	10	4	–	97	–	I.E	[134]
Fe_3_O_4_-CS@BT	Chromium (IV)	20, 50, 60, 100	0.005	25	2	3	62.1 2nd	L	0.99	E.A	[135]
MNPs- polyglycerol	copper (Cu^+2^), nickel (Ni^+^) and Aluminum (Al^+3^)	48	30 mg/L	20	9	2	0.70, 0.45, 0.79 2nd	85 F	98	S.B, M.D	[8]
SBA-15-aminopropyl	Pb (II)	80	1 mg/mL	25	5	4	149.1 2nd	L	0.99	Ch, C	[136]
ZnxFe_3_–xO_4_-NPs	Diclofenac	2.96	0.17	20	–	8 min	–	90	–	O, R	[36]
Fe_3_O_4_-ZnO-chitosan-glutaraldehyde	Brilliant blue	50–250	0.06	60	4	0.58	176 2nd	F	0.97	E, H	[137]
ZIF-8	Arsenic (III) and (V)	10.6	0.5	25	7	24	30.87, 17.51 2nd	L	0.96, 0.98	S.C	[138]
ZrP@1,8-Diaminoctane	U	12	0.06	34	4	–	1.51–1.33 mmol/g 2nd	97 L	0.99	E.A, Ch, C	[139]
HA-O/Fe_3_O_4_	Pb (II), Cu (II), cadmium (Cd) (II), and Ni (II)	10/0.1	1/0.001	25	5.5	–	111.1, 76.2, 71.4, 33.3 2nd	95 L	0.99	E.A	[14]
Fe_3_O_4_-MoO_3_-AC	Ciprofloxacin	5	0.005	25	6 to 8	0.1	47.29 2nd	L	0.99	H, π–π	[140]
Ti3C_2_-MoS_2_	Paraquat	60	0.01	25	7	3	165.384 2nd	F	0.97	E.A	[141]
LDPE/ZnO Blends	Bacteria, fungus, formaldehyde, toluene	1000	20	50	10	0.5	–	B, F: 99.9,F:85.3, T: 82.3	–	D, P, O	[37]
C-Fe/Ni	Ar (V)	1	1	29	6	24	1.176 2nd	87.3 L	0.98	E. R	[72]
ZIF-8@Cu	PFOS	0.1	∼8.0 mg	25	–	24	0.08	98	0.99	E.A, H.I	[142]
PCN-222-MOF	PFOS	500	0.003	25	4.1–5.3	24	2257 2nd	L	0.99	E.A, H, H.I	[143]
Gelatin/zirconium-MOF	Naproxen, ibuprofen	10	-	20	7	12	8.51, 10.23 2nd	79, 77 L	0–94, 0.95	E.A, H, π–π	[144]

^a^L/F: The adsorption isotherm follows the Langmuir or Freundlich models

^b^S.C: surface complexation; H: hydrogen bonding; Ch: chemisorption; E.A: electrostatic attraction; H.I: hydrophobic interactions; π–π: π–π interaction; C: chelation; I.E: ion exchange; S.B: surface binding; M.D: molecular diffusion; O: oxidation; R: reduction; D: disinfection; P: photocatalysis; Co: coordination

*PFOS: Perfluorooctanesulfonic acid

**Fig. 3:**
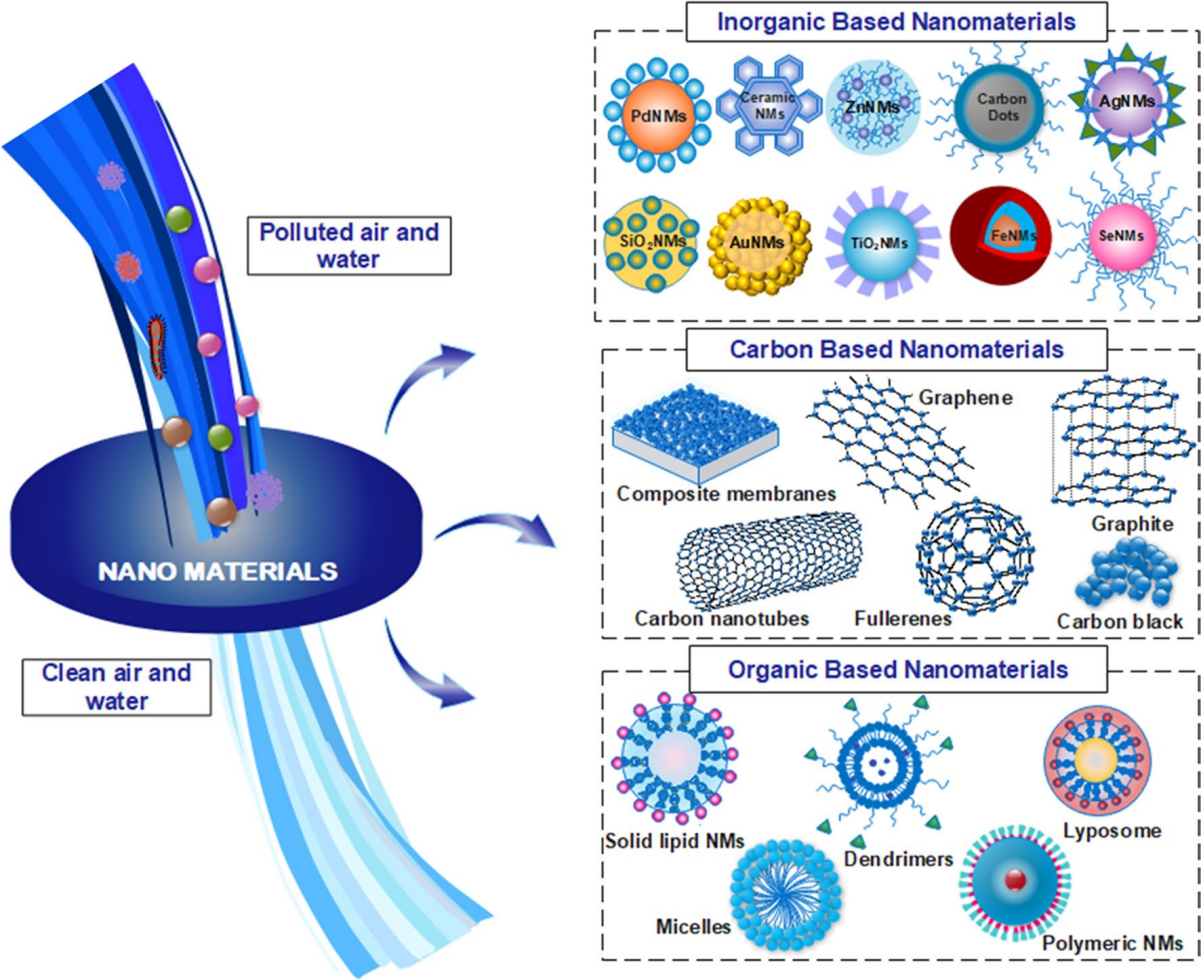
Schematic illustration of diverse types of nanomaterials based on composition employed for air and water pollutants nano remediation. Information was adapted and modify from [69–71]

Metal oxide NMs such as titanium oxide (TiO_2_), gold oxides (Au_2_O_3_), silica (SiO_2_), silver oxide (AgO), tin oxide (SnO_2_), zinc oxide (ZnO), alumina (Al_2_O_3_), manganese oxide (MnO), copper oxide (CuO), nickel oxide (NiO_2_), zirconium oxide (ZrO_2_) and vanadium oxide (V_2_O_5_), cesium oxide (CeO_2_), and magnesium oxide (MgO) encapsulated with carbon-bearing compounds (see Fig. 3) have been extensively studied for the remediation of pollutants [1, 40, 41]. These NMs are known for degrading contaminants through fast reaction rates, stability, availability, non-toxicity, high Brunauer–Emmett–Teller (BET) surface area, and polymorphic structures [42]. They also provide acidic groups necessary for heavy metal binding and protect the encapsulated NMs from aggregation and corrosion [43]. Furthermore, they can inactivate MOs when exposed to ultraviolent (UV) or solar radiation by producing reactive oxygen species such as the hydroxyl radical (OH•), superoxide radical (O_2_•), and hydrogen peroxide (H_2_O_2_) [44]. Recent studies have shown that TiO_2_ NCs have significant reactivity and stability, making them successful in removing heavy metals, eliminating toxic pollutants by gas sensing, purifying indoor air by decomposing them into carbon dioxide (CO_2_) and water (H_2_O), and developing oxidative absorbents for the atmosphere [26, 45]. In addition, the surface of metal and metal oxide base NMs can be easily functionalized using multiple ionic or ionizable groups that may enhance the binding efficiency toward different pollutants. Despite their effectiveness they have inadequate adsorption capacities and low selectivity and are easily deactivated by other aqueous contaminants severely, limiting their effectiveness in removing pollutants. To overcome these limitations, efforts should be made to improve their stability to prevent aggregation, oxidation, and magnetic phase leakage and enhance their recyclability. Additionally, standardizing the synthesis and characterization methods can enhance their efficiency. Furthermore, long-term toxicity studies are needed, focusing on real-life applications to optimize their use. It is essential to consider potential environmental risks and implement appropriate risk management strategies to ensure their safe use. Table 1 shows recent studies with organic and inorganic NMs for remediation of air pollutants from the environment while Table 2 shows recent studies with organic and inorganic NMs to remediate water pollutants.

##### Nano clays

Nanoclays are NMs commonly referred to as layered silicates or nanoclays minerals. They are a type of minerals distinguished by their widespread availability and cost-effectiveness. These minerals are characterized by the presence of layered structures comprising tetrahedral silicate sheets and octahedral hydroxide sheets that give them a plate-like structure [46]. Nanoclays are categorized by their distinct mineralogical compositions, as approximately 30 nano clays variants exist. Their specific attributes render them suitable for diverse applications, each hinging on their unique properties [47]. Among them, montmorillonite (MMT), rectorite (REC), vermiculite (VMT), and kaolinite clay (KC) stands as a prominent choice [48]. They have extensive utility in the enhancement of polymer matrices, to enhance mechanical, thermal, and barrier attributes under high temperature. Polymer chains have the capability to intercalate into the interlayers of clay, facilitating the dispersion of clay within the polymer matrix at a nanometer scale [49]. As the polymer permeates the interstitial gaps between the neighboring nanoclays layers, and the interlayer distance expands, leading to the formation of an intercalated structure [50] Nanoclays find application in diverse fields, including environmental pollution remediation, capitalizing on their attributes like elevated surface area, porosity, and mechanical robustness. However, when synthesizing nanoclays-polymer composites, meticulous attention to each phase is imperative. This is particularly crucial due to the propensity for nanoclays agglomeration and clustering upon introducing substantial quantities into resins. Additionally, an excess input of energy has been noted to trigger premature resin curing, culminating in the brittleness of the final composite products [51].

#### Carbon-based nanomaterials

Carbon-based NMs (CBNMs) are promising adsorbents due to their unique chemical and physical characteristics that enable them to remove organic and inorganic pollutants on a broad scale due to their high surface area and reactivity (see Fig. 3).

##### Carbon nanotubes

Among CBNMs carbon nanotubes (CNTs) have garnered significant attention in recent years due to their unique properties, including high porosity and surface area, high electronic conductivity, and excellent chemical, physical, and mechanical properties [52, 53]. Their highly durable nature and higher adsorption capability make them attractive candidates for various applications. Structurally, CNTs are one-dimensional NMs with cylindrical, sturdy membranous honeycomb lattice structures that effectively capture pollutants [54]. Based on the number of cylindrical shells, CNTs are divided into two groups: single-wall CNTs (SWCNTs) and multi-wall CNTs (MWCNTs) [55]. Numerous studies have investigated the practical applications of CNTs in environmental remediation (Table 2). However, the main obstacle to applying CNTs is their small particle size, difficulty in separation, and poor dispersion in the aqueous phase that significantly hinder their effectiveness. To overcome these challenges, loose clusters/aggregates containing interstitial gaps, grooves, and CNT membranes are widely used for water treatment due to their high adsorption energy sites for organic molecules [56, 57]. Researchers are also exploring techniques such as surface modifications and functionalization to improve the dispersion and separation of CNTs making them more effective for environmental remediation.

##### Graphene based nanomaterials

Graphene-based NMs (GBNMs) are derived from graphene, a two-dimensional carbon allotrope with a honeycomb lattice structure. They include various forms of graphene, such as graphene oxide (GO), reduced graphene oxide (rGO), graphene quantum dots (GQDs), and graphene nanoribbons (GNRs) [58, 59]. Well, GBNMs possess unique properties, such as exceptional mechanical strength, high surface area, high electrical conductivity, and excellent thermal properties, which make them attractive for a wide range of applications, including electronics, energy storage, catalysis, and environmental remediation [40, 43]. The functionalization of GBNMs with various functional groups can also enhance their properties and increase adsorption capacities. Various researchers used graphene based NMs for the adsorption of heavy metals (refer to Table 2). However, it is essential to consider the potential environmental risks associated with GBNMs [60]. To minimize the potential risks associated with GBNMs, strategies such as improving their synthesis, functionalization methods, developing effective monitoring and detection techniques, and establishing regulations for their safe use and disposal have been proposed. Further research is needed to fully understand the long-term effects of GBNMs on the environment and living organisms [56, 57]. Likewise, carbon fullerenes have several unique properties that make them attractive for environmental remediation. With the ability to act as electron acceptors or donors, fullerenes can effectively degrade pollutants through various mechanisms such as photo-induced electron transfer, radical generation, and singlet oxygen generation [61, 62].

##### Carbon quantum dots

Carbon quantum dots (CQDs) are a class of carbon-based NMs that have garnered significant attention due to their promising potential in various environmental remediation applications. They were accidentally discovered while separating other CNTs. These CQDs have special properties like large surface area, customizable fluorescence, and exceptional biocompatibility. These attributes collectively make them highly attractive candidates for a range of applications aimed at addressing environmental challenges thus, CQDs have shown great potential for detecting and removing heavy metals, organic pollutants, and antibiotics from contaminated water sources [63, 64]. CQDs include carbon nanodots (CNDs) and GQDs with fascinating optical properties, such as photoluminescence, and chemiluminescence [65]. They can be synthesized using two methods: the top-down approach and the bottom-up approach. The degree of oxygen content in the oxidized CQDs varies from 5 to 50%, based on the chosen synthesis method. Additionally, they can also detect environmental contaminants through fluorescence quenching and biosensing applications [66, 67]. Two primary categories of fluorescence emission mechanisms have been advanced to explain the characteristics of CQDs. The first category revolves around bandgap transitions resulting from conjugated p-domains, whereas the second category encompasses more intricate origins associated with surface defects present in CQDs [63, 68]. Their low toxicity and biodegradability make them an attractive substitute for conventional remediation techniques. However, a comprehensive exploration is imperative to grasp their complete potential for extensive deployment in environmental remediation endeavors [64, 66].

#### Organic nanomaterials

##### Polymer-based nanomaterials

**Fig. 4:**
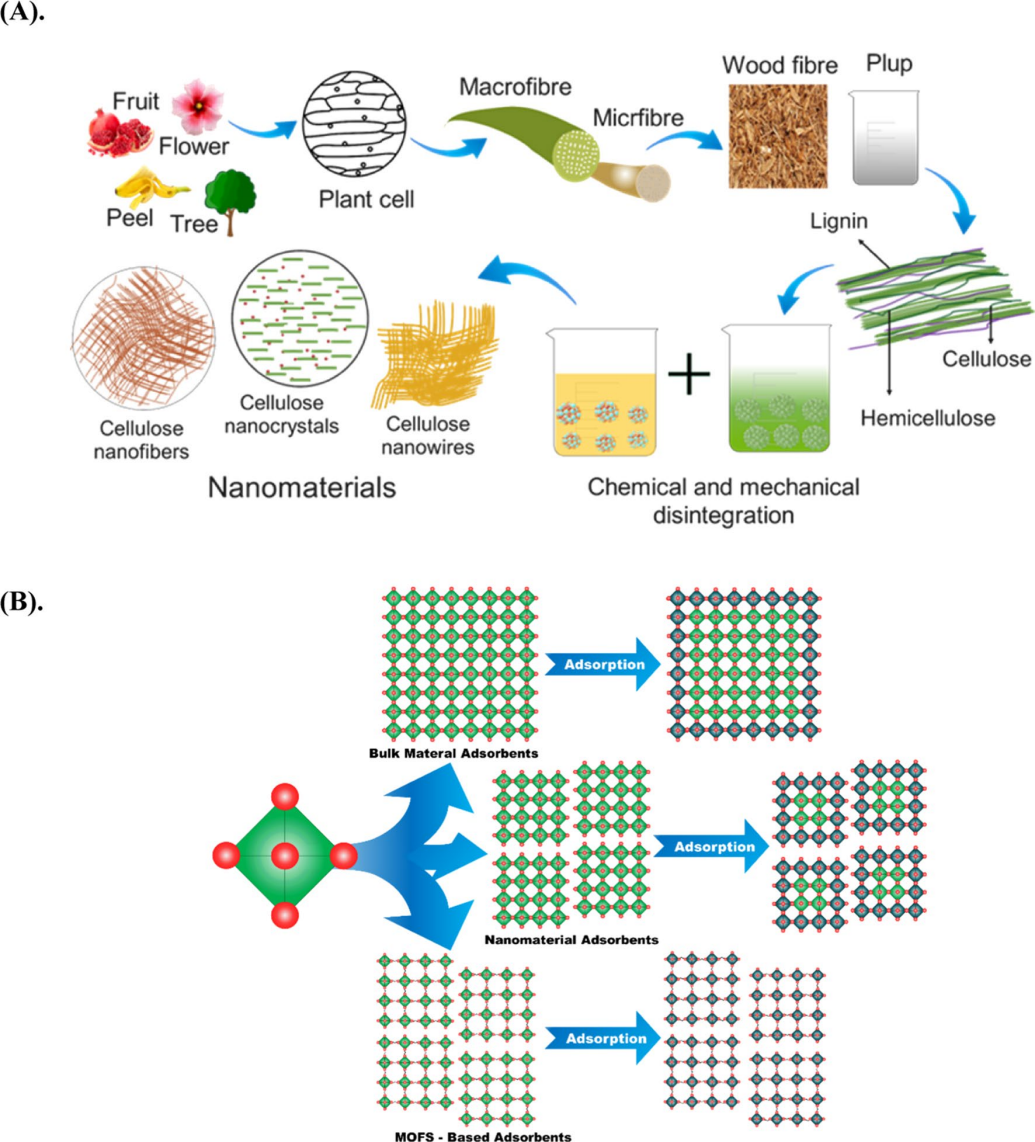
Schematic illustration of **A** schematic illustration of synthesis procedure of green and polymer base NMs, and **B** MOFs and MOF-based materials compared to bulk materials and nanomaterial for adsorption of pollutants. Information was adapted and modify from **A** [96, 97] and, **B** [98]

Among organic NMs natural polymers have been extensively investigated for their potential in environmental remediation owing to their high surface area, poly-functional groups, and superior adsorption and chelating capabilities [72, 73]. Cellulose, hemicellulose, and lignin are the most abundant polymers found in nature and constitute the major components of plant fibers [74, 75]. In addition, easy availability, abundance, and low cost made them attractive bio-based raw materials for NMs synthesis. The synthesis pathway of polymeric NMs is illustrated in Fig. 4A. Cellulose is a linear polysaccharide consisting of repeating D-glucopyranose units joined by glycosidic linkages, making up 30–50% of polymers [76]. It contains functional groups such as methylol and hydroxyl, which make it attractive for inactivating microparticles and metal nanoparticles (NPs) [77, 78]. The presence of six hydroxyl groups per cellobiose unit enhances its adsorption capacity. Increasing the surface roughness of cellulose is necessary for creating composites with better properties [79]. Likewise, hemicelluloses, comprising 20–35% of lignocellulosic biomass, link cellulose and lignin and control cellulose microfibril aggregation [80]. Hemicelluloses contain a variety of neutral sugars and have a β-(1 → 4) backbone like cellulose, and their molecular heterogeneity modulates interactions with cellulose microfibrils through intermolecular interactions and covalent linkages with lignin [81].

The third prevalent polymer is lignin (15–30%), obtained from black liquor from various plant sources [76, 82]. It is an aromatic compound with several functional groups linked by C–C and C–O bonds and has a nanocrystalline heterogeneous structure refer to Fig. 4A [83]. Lignin can be modified through depolymerization to produce newer monomers for developing NMs with inherent properties such as antioxidants, antimicrobial, and UV absorption [84]. Chemical treatments alter polymers properties and enhance organic substance adsorption, with virgin fibers having 20–50 mmol/g capacity and unmodified fibers having 400–1000 mmol/g capacity depending on solute composition and alteration sequence [5]. Polymer-based nanofiber was widely used for water treatment with heavy metals. An overview is shown in Table 2. However, despite having a wide surface area, polymer NMs have limited ability for adsorption of cationic dyes and remaining metals due to a lack of suitable reactive functional groups. To improve adsorbent selectivity the surface polarity and hydrophilicity can be increased by adding additional functional groups. Furthermore, chemical modifications of polymers can improve their hydrophilicity or hydrophobicity, flexibility, microbial resistance, water absorbency, adsorptive or ion exchange, and thermal resistance, ultimately improving their capabilities. Although polymeric NMs require further research, to fully realize the potential of polymeric NMs for environmental remediation.

##### Dendrimers

The term dendrimers, is derived from the Greek word dendra means “tree” and meros means “part” are intricate synthetic polymers characterized by their highly branched, tree-like structure, offer a versatile platform for diverse functionalities and applications. Dendrimers are built either by divergent approach or convergent method [85]. Operating at the nanoscale, dendrimers grabbed attention in the field of drugs and gene delivery. Furthermore, these materials exhibit the potential to revolutionize environmental remediation practices [86]. Notably, dendrimers can serve as efficacious adsorbent to purify water and air by sequestering contaminants. Through strategic functionalization with specific chemical groups like amino, carboxyl, or hydroxyl, dendrimers acquire distinctive surface properties, enabling selective interactions with environmental pollutants such as heavy metals, organic compounds, and dyes [87]. Furthermore, dendrimers demonstrate a propensity to act as carriers for metal chelating agents and other remediation substances, facilitating precise transport to polluted sites for targeted intervention. Moreover, these polymers can be ingeniously engineered to function as sensors, enabling the detection and continuous monitoring of environmental pollution [88]. Amidst their potential, it is paramount to conduct comprehensive research to fine-tune their properties, assess potential risks, and ascertain environmental repercussions. Overall, dendrimers stand as a potent asset in the realm of environmental remediation, presenting a pathway towards a cleaner and safer ecosystem. Their versatile functionalities, ability to selectively interact with contaminants, and potential for tailored delivery make them a promising avenue for driving positive change in our environment [89]. Nonetheless, inadequately delineated chemical structures are a major problem associated with dendrimers. To enhance physicochemical and biological attributes researchers are making attempts through nanotechnology with the aims to elevate solubilization bolster bioavailability, and ultimately address the poorly defined structures [90].

#### Composite nanomaterials

##### Metal–organic frameworks

Metal–organic frameworks (MOFs), or coordination polymers, are crystalline porous materials composed of metal ions or clusters linked by organic ligands to form a 3D structure Fig. 4B [91]. These materials possess various attractive properties, including porosity, tunability, high surface area, and pore size, that facilitate the efficient diffusion of contaminants into the framework [92]. The adaptability of MOF structures is another key advantage, as they can be easily modified by changing the type of metal ion, linkers, and post-synthetic modifications [93]. Moreover, MOFs can be functionalized with chemical groups that selectively adsorb specific contaminants, such as heavy metals, organic pollutants, and gases. Combining MOFs with other materials such as metals, metal oxides, polymers, graphene, and others can create heterogeneous materials that exhibit improved stability over the original MOFs [94]. These MOF-based materials can take different forms, such as hydrogels, aerogels, beads, membranes, and spheres [95]. Due to their unique structural characteristics, MOFs and MOF-based NMs have been extensively investigated for their potential to remediate various air and water pollutants. They have also been studied as catalysts for photo- or electrocatalytic CO_2_ reduction, a rapidly developing field of research [92]. However, MOFs suffer from certain limitations that can impede their practical application. These include structural instability, high production costs, and toxicity risks. Research has been directed towards developing functional MOF-based NMs to overcome these challenges and optimize the potential in various applications. Future investigations could explore the development of stable production methods, cost-effective production techniques, alternative ligands and metals, and hybrid materials to unlock the full potential of MOFs in environmental remediation and other areas of use.

##### Nanocomposite membranes

**Fig. 5:**
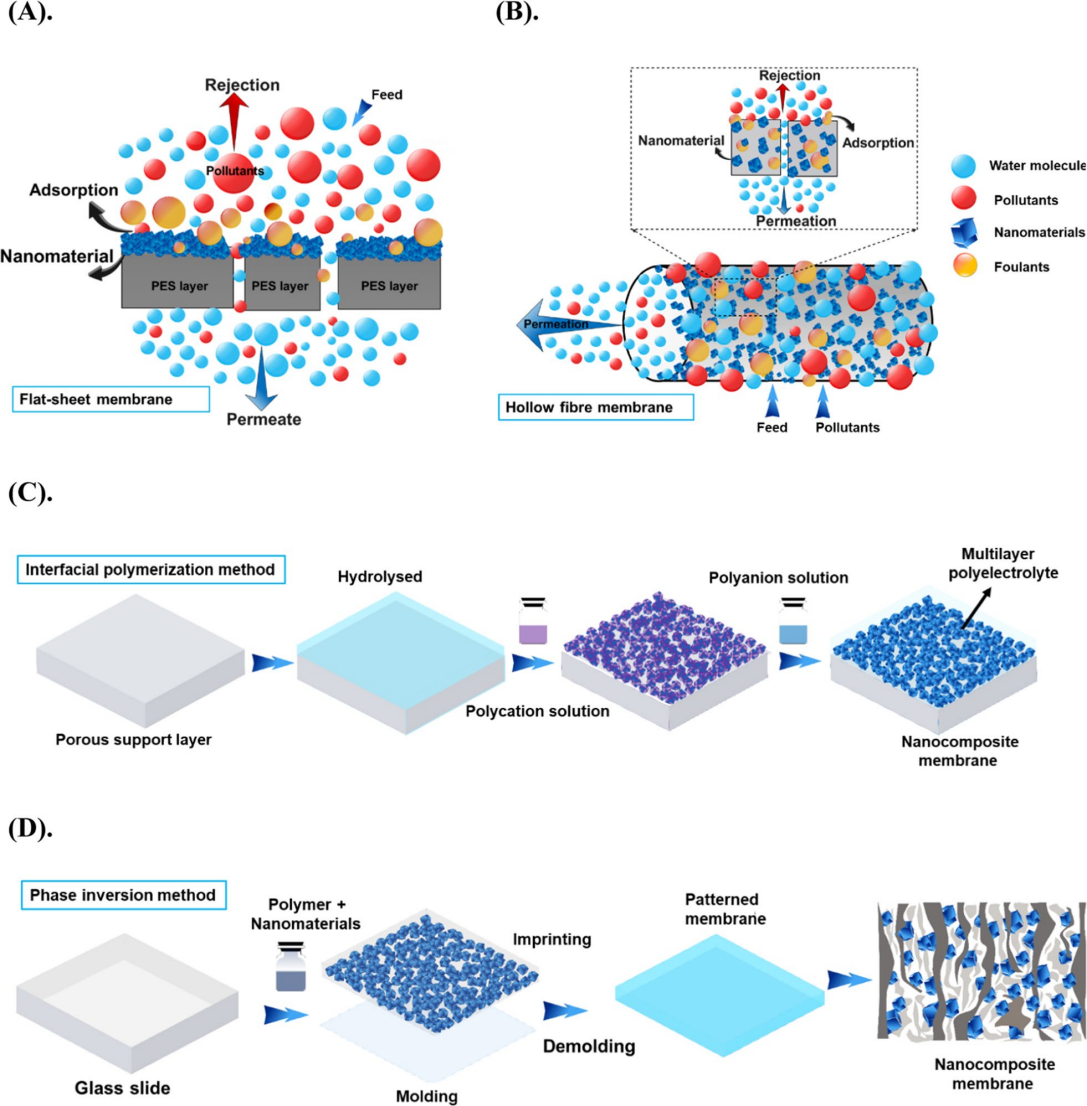
Schematic illustration of nano composite membranes **A** flat sheath, **B** hollow fiber nano composite membranes, **C**, **D** synthesis steps of nanocomposite membranes, and **E** nano sensors for air and water pollutants remediation. Information was adapted and modify from **A** [105] **B** [106] **C** [107] and, **D** [108]

Nano-composite membranes (NCMs) are a new breed of adsorbents and membranes made of polymeric and non-polymeric materials, such as metals and ceramics [5]. These membranes have numerous applications in environmental remediation and pollution control because of their superior properties, such as improved mechanical, thermal, and chemical properties, increased hydrophilicity, and effective removal of pollutants such as dyes, heavy metals, and antibacterial capabilities [59, 99]. The NCMs encompass several types, such as inorganic–organic hybrid, polymeric, ultrafine, CNT, polymeric, and MOF membranes. These membranes can be created by incorporating various types of nanoparticles (NPs), including TiO_2_, Al_2_O_3_, ZrO_2_, CNTs, Ag, SiO_2_, MOF, covalent organic framework (COF), and NZVI, into the polymeric membrane matrix as shown in Fig. 5A–D. This incorporation leads to a notable improvement in the performance of the NCMs, such as enhanced functionality, thermal stability, and microbial inactivation properties, while maintaining the structural integrity of the membrane [100]. The fabrication process of NCMs typically involves mixing the polymer matrix and inorganic NPs together, followed by casting or electrospinning the mixture into a membrane as shown in Fig. 5C and D. The properties of the membrane can be fine-tuned by varying the type, size, and concentration of NPs and polymer matrix [101, 102]. Interfacial polymerization and phase inversion methods are two widely used techniques in the fabrication process of NCMs. In interfacial polymerization, the reaction occurs between monomers present in different phases at the interface between them, which leads to a thin, uniform polymer layer on the surface of NPs. This method is beneficial when synthesizing inorganic–organic hybrid and MOF membranes. On the other hand, phase inversion methods involve converting a homogeneous polymer solution into a porous membrane structure by manipulating the thermodynamics of the polymer solution refer to Fig. 5C and D [53, 103]. This process includes dissolving the polymer in a suitable solvent, casting it onto a substrate, and extracting or evaporating the solvent, forming a porous membrane structure. This method is well-suited for fabricating polymeric and CNT membranes [104]. Other techniques, such as layer-by-layer assembly, sol–gel or chemical vapor deposition can also fabricate NCMs with specific properties. Although the use of NCMs deteriorates a wide range of pollutants, however, they tend to leach out and aggregate, especially if they are grafted on a membrane without surface protection, which could make the process more difficult and low contaminants degradation. Thus, to address the disadvantages, future research can focus on improving selectivity, stability, and durability of NCMs. Furthermore, to exploring new types of NMs and advanced characterization techniques, and developing sustainable and cost-effective fabrication methods for large-scale production.

##### Nano sensors

Nano sensors empowered by NMs allow precise detection and remediation of environmental pollutants at the nanomolar to sub-picomolar scales. Nano sensors are energy converters at nanoscales that can recognize and detect chemical, machinal, and physical phenomena at the nanoscale in the environment and give an electrical or optical signal as output. Nano sensors comprise a specificity-enhancing recognition component and a signal transmission technique that enable them to confirm the presence of an analyte [17]. Nano sensors are susceptible, with high detection power and capacity to monitor multiple tasks at once, and they can easily suspend in the air for a long time to collect information through their wireless and send it to a central base. With the use of nano sensors, air pollution has been effectively controlled. Nano sensors of CNTs, SnO_2_, Pt, Cu, Ag, and CQDs have been reported to remove toxic gases and heavy metals from the environment. Furthermore, nano sensors of NCs coated with gold (Au), silica oxide (SiO_2_), zinc (Zn), and lead (Pb) have been reported to detect heavy metals in drinking water.

## Available pathways to synthesize nanomaterials

### Traditional pathways

**Fig. 6:**
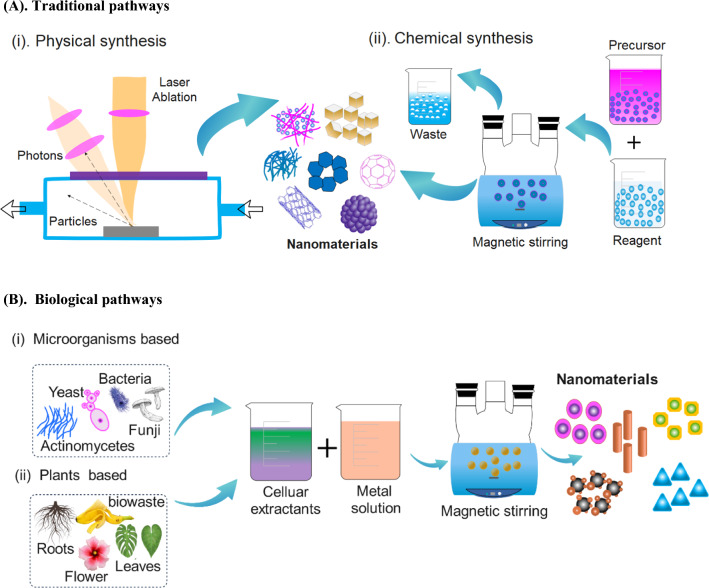
Graphical illustration of general procedures for nanomaterial synthesis. **A** traditional pathways and, **B** biological pathways. Information was adapted and modify from [151, 152]

Traditionally, NMs can be synthesized in two different ways chemical and physical synthesis pathway. Chemical synthesis includes liquid and gas phase pathways, as shown in Fig. 6A. Among them liquid phase methods include colloidal methods, precipitation, sol–gel, coprecipitation, solvothermal, water–oil microemulsions, hydrothermal methods, chemical reduction, polyol approach, and radiolytic method, while gas phase includes pyrolysis and inert gas condensation method [145]. Furthermore, ionotropic gelation and microemulsion methods are also used to synthesize NMs using polyelectrolytes, enzymatic treatment, ultrasonic, acid hydrolysis, chemo-mechanical therapy, and nanoprecipitation. Physical synthesis utilizes various physical processes to create structures with unique properties and characteristics. Similarly, the two different ways of synthesis as already discussed in subheading "Carbon quantum dots" (top-down and bottom-up strategy). Top-down strategy utilizes mechanical processes such as milling, grinding, or etching to reduce bulk materials into smaller particles. In contrast, bottom-up strategy involves assembling smaller units, such as atoms, molecules, or NPs, into larger structures. Production techniques such as lithography, etching, and exfoliation are used in top-down strategy, while wet chemical precipitation, sol–gel, chemical vapor deposition, hydrothermal, sputtering, template growth, electrospinning, and atomic layer deposition are used in bottom-up techniques. Traditional pathways present limitations and disadvantages, such as high energy requirements, special equipment, and high costs [15, 132]. These challenges can be overcome by introducing functional groups to the surface of NMs in various ways to improve their effectiveness, selectivity, and sensitivity. These methods include creating chemical bonds between the modifier and NMs surfaces or physically adsorbing the modifying species to the NM surface [132]. However, the chemicals used in these processes can be corrosive, toxic, and flammable, posing significant environmental problems [104]. Furthermore, the traditional pathways are toxic and costive; therefore, it is foreseen that green and polymer base NMs will emerge as a hotspot for future research studies, especially on industrial-scale applications. Therefore, it is recommended to adopt simple and reliable alternative methods such as synthesis and use of green based NMs for NMs synthesis.

### Biological pathways

To achieve more sustainable and eco-friendlier NMs production, scientists have started exploring the use of less hazardous and toxic chemicals in the manufacturing process [146, 147]. Green and biobased NMs production has gained attention as a promising alternative to molecular solvents due to its potential to remediate environmental pollution cost-effectively and sustainably. This is achieved by using non-toxic precursors and environmentally friendly solvents during the synthesis process, which minimizes the production of harmful byproducts [148]. It involves extracting various biological materials, including bacteria, fungi, algae, yeast, green extracts, proteins, polysaccharides, nitrates reductase, coenzymes, biosurfactants, and hemicellulose biomass to produce a wide variety of sizes and forms of NMs [16] shown in Fig. 6B. Some of the most important environmental features of biologically synthesized NMs are biodegradability, sustainability, and environment friendly, as they rely on renewable resources and reduce the use of hazardous chemicals [72]. Plants raw materials possess various functional groups with reducing capabilities and phytochemicals that can serve as coating agents to stabilize the NMs and facilitate multidimensional absorption. Valuable plant materials such as leaves, roots, flowers, and fruit have been investigated for their potential to produce NMs [149, 150]. For instance, plant leaf extracts contain a range of phytochemicals, including alkaloids, tannins, flavonoids, carotenoids, vitamins, minerals, amino acids, sterols, glycosides, alkaloids, flavonoids, phenolics saponins, and phenolic compounds which act as reducing agents to eliminate toxic chemicals, MOs, and hazardous pollutants in environment [83, 113]. Compared to chemically synthesized counterparts, biologically synthesized NMs offer a higher potential for sustainable growth and environmental remediation. Recently biologically synthesized NMs have widely reported holding significant potential for effectively mitigating environmental pollution and promoting sustainable development (refer to Table 3). Future studies should prioritize investigating the scalability, cost-effectiveness, and safety considerations of biologically synthesized NMs to enable their widespread commercialization and adoption in industries.

**Table 3:** Summary of experimental conditions, adsorption kinetic, removal efficiencies and mechanisms of different organic and inorganic NMs for environmental pollutants remediation

Nanomaterials	Pollutants	Experimental conditions					q_max_ (mg/g) Ads kinetics	Removal efficiency (%) (L/Fa, A)	R^2^	Mechanism	References
		Sorbent mg/L _initial_	Adsorbent dose (g/L)	Temp ( ͦ C)	pH	Time (h)					
Fe–Ni-Eucalyptus leaf extract	Ar	0.8 g	0.4–4	20	6	24	2^nd^	87.3 L	0.99	S.C, E.R	[72]
ZnO-Banana peel extract	Basic Blue 9, crystal violet, and cresol red	30	2.0 × 10^–5^ M	–	12	1.5	–	100	–	P	[153]
Fe (nZVI)-pomegranate peel coated activated carbon	Amoxicillin	50	1	20	5	0.5	40.28 2nd	97.9, L	0.99	–	[154]
Activated carbon-Neem (Azadirachta indica) leaf extract	Ciprofloxacin	10	1.2	30	4	3	2nd	89.5 L	0.99	I.E, π–π, H	[155]
Activated carbon-pomegranate husk	4-Chlorophenol	20	0.4	20	6	2	26.18, 24.78, 2nd	100 L, A	0.99	H	[156]
Chitosan-iron-activated carbon-orange peel	Nitrate	50	0.1	20	2	3	263.15 2nd	99.59 L	1	I.P.D	[157]
Fe_3_O_4_-Azardica indica leaf extract	Methyl blue, 2-Nitroaniline	140, 0.05	5	21–23	6.3	2.67	–	99.96, 83	–	R	[158]
CuO-Moringa oleifera extract	Nitrate	100	0.5	25	2	24	7.98 1st	60 L	0.99	E.A	[159]
TiO_2_-Jatropha curcas L. leaves extract	Chemical oxygen demand (COD), Cr VI	5000	6.8, 3.2 mg/L	59	UN	5	–	82.26, 76.48	0.95	P	[160]
Cu_2_O-lignin	Bacteria	10 μL	90 μL	37	7	24	–	100	–	D	[82]
Cu_2_O- astragalus, rosemary and mallow	Pb	0.030	0.2	25	7	24	41.1, 39.8, 32.4, 1st	88.4, 84.9, 69.6, L	0.99	Co, C	[149]
Activated carbon- pomegranate peel- nZVI	Amoxicillin	10 mL	1.5	25	5	0.5	40.28 1st	83.54 L	0.99	I.P.D	[154]
FeNPs-Korla fragrant pear peel extracts	Cr	10	0.02	55	5	2	46.6, 1st	99.1, L	0.97	R, C.P	[52]
ZnO-Syzygium cumini plant leaves extract	Methylene blue	2 mg/50 mL	0.5	–	8	2	1st	91.4		P	[161]
FeNPs-(Eucalyptus leaf extracts	Ammonia, Phosphate	10	7	30	6.35	0.5	3.47, 38.91 2nd	43.3 NH_4_^+^, 99.8 PO_4_^3−^, L	0.95–0.97	Ch	[150]
AgNPs-Ficus Benjamina leaf extract	Cd	50–100	0.05	25	5	0.67	2nd	F	0.97	–	[162]
Fe_3_O_4_-wheat straw	Hg°	40	1	25	6	0.75	101.01 2nd	98 L	0.99	E.A	[163]
ZnO-Artemisia absinthium	Cr (VI)	25	0.25	25	4	2.5	315.46 2nd	99 L	0.99	E.A	[164]
α-Fe_2_O_3_-Aloe vera leaf extract	Ar (V)	10	0.5	20	6	12	7.95 2nd	98, L	0.99	Ch	[165]
Cu0 + Ag)@Bentonite-P. guajava leaves	Amoxicillin, Sulfamethazine	2.0	0.3	25	3	2	1st	84 and 74	0.98	P	[166]
CuO/NiO-Capparis decidua	Lambda-cyhalothrin	20	0.002	25	7	3	1st	99, 89		R, O, P	[167]
Rice straw biochar-alginate beads	Per- and polyfluoroalkyl	0.1	1	–	7	16	1.572 2nd	87–99 F,L	0.99, 0.97	Ch	[168]
Fe_2_O_3_ -black tea	Ametryn	30.0 μg/L	2.5 g/L	20	7	0.5	2nd	F, L, T, D	0.98	L.F.D	[169]
Fe_3_O_4_-office paper (cellulose fibre)	Cobalt oxide	100 mg L^−1^	100 mg L^−1^	25	5	48	1567 2nd	L	0.94	Ch, E.A	[75]

^a^L/F: The adsorption isotherm follows the Langmuir or Freundlich models

^b^S.C: surface complexation; H: hydrogen bonding; Ch: chemisorption; E.A: electrostatic attraction; π–π: π–π interaction; C:chelation: I.E: ion exchange; I.P.D: inter particle diffusion: R: reduction: D; disinfection; Co: coordination; P: photocatalysis; O: oxidation; L.F.D: liquid film diffusion; C.P: coprecipitation

## Mechanisms for pollution remediation using nanomaterials

The fundamental principles utilized in remediating environmental pollution encompass three main approaches: physical, chemical, and biological treatments. Each approach operates through specific reaction mechanisms. Physical treatment involves adsorption and radiation mechanisms, chemical treatment employs oxidation and reduction mechanisms, while biological treatment includes disinfection using aerobic and anaerobic microbes, and enzymatic processes [170].

### Adsorption

**Fig. 7:**
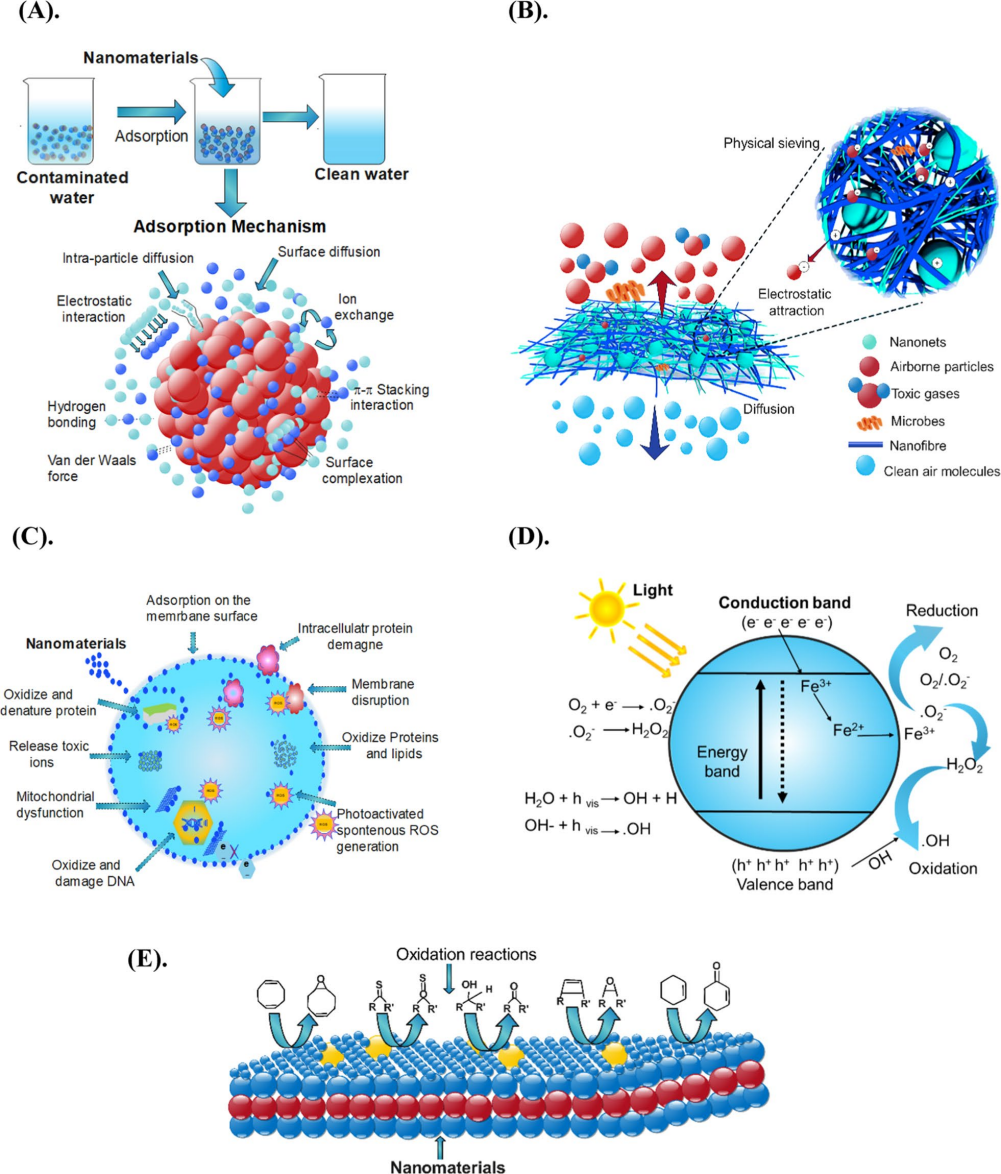
**A** adsorption mechanism **B** filtration mechanism, **C** disinfection mechanism, **D** photocatalytic mechanism, and **E** oxidation mechanism of different pollutants with NMs. Illustrations were adapted for modification from [5, 35, 41, 175, 189, 198]

Adsorption is a widely used technique to eradicate air and water pollutants. It is an excellent practical approach and lacks toxic by-products to mitigate organic and inorganic pollutants [40]. Adsorbents are developed using altered, unaltered and enhanced NMs encompassing polymers, activated carbon, MOF, molecular sieves, zeolites, and other economically viable substances. The selection of a specific adsorbent hinges primarily upon its inherent adsorption capacity and the materials intrinsic affinity for the targeted compound [171, 172]. The adsorption process involves separating chemicals from one phase and concentrating them on the surface of another adsorbent material [12]. Adsorption occurs in three steps: (1) interaction of an adsorbed species with an absorbent in interface with a liquid state and a solid phase of different compositions at a constant temperature and pressure over a specific duration of time. (2) The adsorbent and liquid layer are separated after the reaction, and (3) the adsorbent material in the supernatant liquid state and any pressurized fluid phase are measured [15, 21]. The mechanism is presented in Fig. 7A. The adsorption rate of adsorbate is primarily influenced by two crucial factors, namely adsorption isotherms, and kinetics, which represent the adsorbent's adsorption effectiveness and establish the adsorption parameters. Thus, a thorough grasp on thermodynamic and kinetic aspects is necessary. The Langmuir and Freundlich isotherm models and the pseudo-first order, pseudo-second order, and intraparticle diffusion models are key isotherm and kinetic models commonly used to describe the adsorption process [83]. Through kinetic analysis, we can determine the residence time required for completion of adsorption reaction, and kinetic information aids in sizing the adsorption equipment appropriately. It also evaluating the efficacy of fixed-bed systems or other flow-through setups [172]. Adsorption is widely recognized as the primary mechanism for removing heavy metals, dyes, and other pollutants. Previous studies have proposed four distinct adsorption mechanisms: ion exchange, electrostatic adsorption, surface physical adsorption, and complexation/chelation [40, 75]. The adsorbed substance is classified as either physisorption or chemisorption. Physical or physisorption usually adsorbate adheres through van der Waals (weak intermolecular) interactions. In contrast, chemisorption involves the formation of strong chemical bonds between the adsorbate and the adsorbent surface [8, 173]. Electrostatic interactions, π-π and hydrophobic interactions, acid–base interactions, and van der Waals interactions all play a crucial role in determining the efficacy of nano adsorbents in absorbing organic compounds, complex compounds, and heavy metals. The NMs surface offers many active sites for interaction with various chemical species due to its small size, high surface area, and surface multi-functionalities [10, 174]. Many factors influence the adsorption efficiency of hazardous pollutants, e.g., pH, operating temperature, amount of adsorbent, suspended particles, surface charge, concentration of adsorbent and adsorbate and contacting time (discussed in our previous study) [175]. Each of these factors needs meticulous optimization in order to determine efficiency of absorbent. In recent studies, the adsorption capacity onto NMs was reported to strongly influenced by the pH of the solution specifically, increase in pH because it was observed to promotes adsorption when the surface of the adsorbent has a negative charge [15, 174]. The underlying mechanisms of this process involve molecular diffusion and surface binding. Conversely, when the pH is lower, and the surface of the adsorbent becomes positively charged, the adsorption of pollutants slows down due to reduced electrostatic attraction. It is crucial to control solution conditions for fine-tuning adsorption efficiency and enhance adsorbents effectiveness. As suggested in literature, incorporating NMs by blending them with adsorbents can improve the attraction between the desired substances and the adsorbents. These insights can enhance the design and optimization of nano adsorbents for more effective removal of pollutants from contaminated resources [43, 100].

### Membrane filtration

Membrane filtration serves as a fundamental technique for segregating pollutants using porous materials or membranes that selectively allow desired particles or substances to pass while retaining others [176]. In recent times, membrane filtration has garnered significant interest for being a cost-effective and highly efficient method. The filtration process is influenced by factors like particle size, surface charge, solute hydrophilicity, and shape. Traditional membrane filters are typically crafted from petroleum-derived polymers like polyethylene (PE), polypropylene (PP), and glass fibers [177, 178]. The filtration mechanism in membranes occurs through physical capturing processes such as sieving, inertial impaction, interception, and diffusion. The four straightforward physical capturing processes of sieving, inertial impaction, interception, and diffusion are primarily used to fulfill filtration functions [179]. The mechanism is presented in Fig. 7B**.** Brownian motion can describe the filtration mechanism of nanometer-sized particles, but it is unlikely to be a sufficient mechanism for filtration on its own. Brownian motion causes some particles to diffuse toward the surface and deposit as they pass on both sides of the membrane filter. This happens when particles pass at a distance for collision by inertia or interception [178–180].

However, membrane filter properties can be fine-tuned using fabrication methods involving the integration of NMs and polymers. These modifications play a pivotal role in enhancing membrane performance and mitigating fouling. By incorporating or blending of NMs like Ag, SiO_2_, TiO_2_, ZnO, CNTs, polymers base NMs, metal oxides, halloysites, and more, membrane characteristics can be adjusted. This infusion introduces functional groups and heightens hydrophilicity and adsorption capabilities, thereby bolstering their efficacy in rejecting contaminants [179]. This leads to improved removal of MOs and noxious pollutants. Notably, photocatalytic NMs, in particular, exhibit substantial promise in eradicating toxic pollutants through photo degradation [180, 181]. However, they only target solid particles and cannot capture and remove other toxic contaminants, and when disposed of, might result in secondary pollution. Recent research is focusing on creating hybrid membranes with incorporation of NMs. Furthermore, modification of membranes was reported to enhance removal of harmful pollutants [182, 183]. The suggested modification enhancements reported in literature involve surface modification to improved selectivity and hydrophilicity and interfacial polymerization to enhance strength, and chemical/thermal stability while also offering features like anti-fouling properties, self-cleaning ability, selectivity, and the capacity to combat microbes. They provide a high flow rate under low pressure conditions and allow for easy recovery and reuse of the NMs [176–178].

### Disinfection

Well, NMs have been used as disinfectants due to their non-specific mode of action to remove organic and inorganic contaminants and inactivate various types of MOs, such as viruses, protozoa, and bacteria [13]. Conventional disinfectants, such as chloramines, ozone, chlorine, chlorine dioxide, and chlorine gas, can effectively prevent microbial growth, but they have short-lived reactivity and can present problems since they can produce toxic, carcinogenic disinfection byproducts (DBPs) [184, 185]. NMs successfully overcome drawbacks that prevented the viability of conventional disinfection and have established themselves as effective disinfecting agents in environmental remediation. NMs exhibit such behaviors due to their improved interfacial charge separation, increased surface area, solution chemistry, and transport behavior, which provide more active sites and prevent DBPs production, thus increasing disinfection [186]. Additionally, NMs are highly stable and can remain active for extended periods, reducing the need for frequent reapplication [187]. Many NMs have been proposed to inhibit the growth of microbes in air and water, including CNTs, chitosan, fullerene, TiO_2_, MgO, ZnO, AgO, and zerovalent NMs.

The disinfection mechanism operates through the adherence of NMs (such as ZnO + , Ag + , Ti +) to the lipopolysaccharide layer within the external cell walls of target microbes. This interaction induces oxidative stress (ROS), which subsequently degrades the peptidoglycan layer, triggering peroxidation of the lipid membrane. Consequently, oxidation extends to the membrane proteins, inducing alterations in membrane characteristics that prompt the release of cations [188]. Even at low concentrations, these cations initiate a biological response. They incapacitate bacterial respiratory enzymes by binding to functional groups like thiol groups in proteins, inducing damage to microbial cells by disrupting DNA, oxidizing lipids, peroxidizing proteins, and impeding cellular respiration [189]. This sequence ultimately leads to the malfunction of the cellular respiration process and peroxidation of polyunsaturated phospholipid components in the cell membrane, culminating in cell death [186, 188]. Moreover, the interactions between cations and DNA can inhibit replication and create structural alterations in the cell envelope [104]. The mechanism is presented in Fig. 7C.

### Photocatalysis

Photocatalysis has emerged as an effective and environmentally friendly process for the degradation of persistent organic pollutants (POPs), endocrine disrupting chemicals, heavy metals, insecticides, acetaldehyde, nitrogen oxides (NOx), sulfur oxides (SOx), ammonia (NH_3_), carbon monoxide (CO), mercury (Hg), volatile organic compounds (VOCs), and polycyclic aromatic hydrocarbon (PAHs) [18, 117, 167, 190]. This process enhances chemical reactions that transform toxic air pollutants into non-toxic gases and in their powder form often result in complete mineralization. However, it still has drawbacks such as poor recovery and secondary contamination [103, 190]. The process mechanism involves the use of a light source to activate a wide-bandgap semiconductor materials, such as TiO_2_, ZnO, SnO_2_, cadmium sulfide, and tungsten trioxide, in the presence of water, causes electron separation, and develops an electron–hole pair [5, 41]. The photocatalysis mechanism entails several steps starting with photoexcitation that triggers a series of oxidative and reductive reactions on the surface of the photocatalyst upon exposure to an appropriate wavelength of light (often with photon energy (hv) more significant than or equal to the band gap energy) [149, 191]. Subsequently, the electrons diffuse across the photocatalyst's interface and interact with the surroundings, leading to reductions and oxidations [192, 193]. Photocatalytic oxidation involves the use of a catalyst activated by an energy source to facilitate reactions that rely on the production of highly reactive radical species such as OH^•^ and ^•^O_2_^−^, which are potent oxidizing agents that are generated through a reaction between photogenerated electrons and molecular oxygen and between photogenerated holes and water, that non-selectively destroys all organic contaminants [92, 194]. The mechanism is presented in Fig. 7D. The photocatalysis approach can be divided into two categories: homogeneous photocatalysis (photo-Fenton reaction), which responds up to a light wavelength of 600 nm, and heterogeneous photocatalysis (Fenton reaction), which does not entail any light irradiation [35]. In the context of large-scale applications, the photocatalytic performance of nano catalysts may be hindered by the presence of complex chemical mixtures and prolonged irradiation periods. Several strategies have been developed to overcome these limitations to enhance nano catalysts photocatalytic performance. These include introducing anionic and transition metal dopants and tailoring the surface properties of the nano catalysts. Dopants such as sulfur, fluorine, carbon, and nitrogen can modify the electronic structure of the nano catalysts and increase the number of active sites, resulting in higher efficiency. Nanofibers or nanotubes can also increase the surface area, while plasmonic materials such as Au or Ag NPs can enhance light absorption [103, 190]. Moreover, the incorporation of blended photocatalysts with supportive materials is a well-regarded approach in the literature. This technique not only enhances the recoverability of photocatalytic agents but also mitigates the potential for secondary contamination. As a result, it facilitates the attainment of desirable attributes such as recyclability, reusability, and an extended lifespan of the photocatalytic components.

### Oxidation

Organic and inorganic compounds can be oxidized by using NMs that produce active oxygen species such as superoxides and hydroxyl radicles. Commonly used metal oxides for oxidation are MnO_2_, V_2_O_5_, CuO, TiO_2_, ZrO_2_, and CeO_2_ [26, 41]. Oxidation is a process in which oxygen-containing groups are created on NMs by splitting the reaction media and is typically carried out using an oxidizing agent e.g., H_2_O_2_, potassium permanganate (KMnO_4_), sodium hypochlorite (NaOCl) and one or more inorganic acids such as nitric acid (HNO_3_) and sulfuric acid (H_2_SO_4_) in a refluxing state [41, 192]. The reaction mechanism of oxidation is still not fully understood, and the relevance of the particle size and surface morphology is still under debate. The mechanism of oxidation is presented in Fig. 7E. It was stated in literature that the mechanism of oxidation produces ROS such as (^•^OH, ^•^O_2_^−^, SO_4_^•−^), and singlet oxygen (^1^O_2_) to improve the degradation of organic contaminants. For the decontamination process to take place, the production of these radicals need to be adequate [195, 196]. Different metal oxide NMs can remove gases and metals from the air using liquid-phase, gas-phase, and combined-phase oxidation methods that can be achieved by heating or plasma treatment in the presence of oxygen gas [54]. Well, liquid-phase oxidation is a more common method for removing metals and functionalizing products while advanced oxidation processes (AOP) are also a significant component of liquid-phase oxidation. The commonly used gaseous oxidants include ozone (O_3_), chlorine dioxide (ClO_2_), and non-thermal plasma [8, 100, 169]. Transitional metals such as (Fe, Co, Ti, Zr, V, Mn, Cu, and Ce) are widely used to simultaneously remove toxic gases and heavy metals from the air by catalytic oxidation. These metals have large holes and favor redox reactions for electron transfer, resulting in lower reaction activation energy. Metals can be doped or impregnated onto or into the substrate material to enhance the surface area of the catalyst and increase its reactivity in both oxidation and reduction of heavy metals and gases, improving their resistance to gases and water [26, 197]. So far, various oxidants are commonly used to eliminate harmful gases from the atmosphere, including chlorine-based and sulfur-based compounds, O_3_, and H_2_O_2_ [26]. Some oxidative absorbents are highly reactive and safe to use, with no toxicity, such as ClO_2_, hypochlorite (ClO^−^), chlorite (ClO_2_^−^), and chlorate (ClO_3_^−^). Additionally, persulfate (PS, S_2_O_2_^−^) and permonosulfate (PMS, SO_2_^−^) are also recommended for their ease of storage and transport [18, 197]. The reaction mechanism of oxidation is still not fully understood, and the relevance of the particle size and surface morphology is still under debate. The mechanism of oxidation is presented in Fig. 7E. It was stated in literature that the mechanism of oxidation usually takes place between chemisorption of CO and dissociative adsorbed oxygen. For the decontamination process to take place, the production of the radicals needs to be adequate.

## Future strategies for appropriate eco-safe nano remediation

Although NMs are widely used for the remediation of environmental pollutants, they may still have unintentional adverse effects on human health and the environment when released in massive quantities and accumulate in the food chain. Therefore, it is crucial to develop sustainable technologies that can reliably remediate pollutants while minimizing the risk to human health and the environment. The efficient application of NMs for remediating toxic pollutants requires careful consideration of their advantages and disadvantages and their dispersion and retention properties. Each type of NMs has unique advantages and disadvantages, making it challenging to determine which is best suited for environmental remediation.

Chemically synthesized NMs have been proven harmful to human health and the ecosystem, making it necessary to develop sustainable, effective, and powerful NMs for environmental remediation. While polymer and green-based NMs are still being studied on a laboratory scale. Scientists have been using supporting materials such as plant waste and polymers to modify the structure and composition of NMs to boost their efficacy. Low-cost and readily available sources such as bone char, charcoal ash, fly ash, rice hull ash, and pomegranate cover can be used as absorbents and supporting materials to enhance efficiency and minimize the drawbacks of chemically synthesized NMs [6, 199, 200]. Comparing the adsorption capacity of each NM is difficult as the parameters and adsorbates employed are always different. However, various NMs have been found to have strong metal ion sorption abilities. Furthermore, research is needed to coat NMs, allowing them with surfactant to reach pollutants and destroy themselves after performing their job, such as coating them with a surfactant. The use of NMs for environmental remediation must be approached strategically, considering their impact on human health and the environment, to ensure appropriate and eco-safe remediation. In addition, the utilization efficiency, engineering investments, and operational expenses associated with each type of NM must be compared to determine the most suitable approach.

## Conclusions

This systematic review highlights the immense potential of NMs for mitigating environmental pollutants. NMs offer a promising avenue for addressing environmental pollution, displaying potential advantages over conventional methods. This review covers the following aspects. Discussion on both conventional and advanced pollutant removal techniques, emphasizing the potential of NMs in this context. Exploration of NMs types, encompassing inorganic variants (metal and metal oxides, nanoclays), carbon-based, graphene based, carbon quantum dots, organic counterparts (polymer-based, and dendrimers) and nano composites (MOFs, NCMs, and nano sensors). Evaluation of diverse synthesis pathways, encompassing traditional methods (chemical and physical) as well as biological synthesis routes for NMs. Elaboration on reaction mechanisms facilitated by NMs in pollutant removal, encompassing adsorption, filtration, disinfection, photocatalysis, and oxidation and provision of strategic insights for future research strategies.

## Data Availability

The authors would like to confirm the availability of data and materials associated with the manuscript and fully support the importance of data transparency and reproducibility in scientific research.
